# Goldfish Leptin-AI and Leptin-AII: Function and Central Mechanism in Feeding Control

**DOI:** 10.3390/ijms17060783

**Published:** 2016-05-30

**Authors:** Ai-Fen Yan, Ting Chen, Shuang Chen, Chun-Hua Ren, Chao-Qun Hu, Yi-Ming Cai, Fang Liu, Dong-Sheng Tang

**Affiliations:** 1 College of Medicine, Foshan University, Foshan 528000, China; yanaifen@mail3.sysu.edu.cn (A-F.Y.); 1115fang@sina.com (F.L.); tangdsh@163.com (D.-S.T.); 2 CAS Key Laboratory of Tropical Marine Bio-resources and Ecology (LMB), Guangdong Provincial Key Laboratory of Applied Marine Biology (LAMB), South China Sea Institute of Oceanology, Chinese Academy of Sciences, Guangzhou 510301, China; rosemary166@sina.com (C-H.R.); hucq@scsio.ac.cn (C.-Q.H.); cym052065@163.com (Y.-M.C.); 3 South China Sea Bio-Resource Exploitation and Utilization Collaborative Innovation Center, Guangzhou 510275, China; 4 School of Biomedical Sciences, Li Ka Shing Faculty of Medicine, University of Hong Kong, Hong Kong, China; chenss@connect.hku.hk

**Keywords:** leptin, goldfish, methylotrophic yeast, feeding control, gene regulation, appetite regulators

## Abstract

In mammals, leptin is a peripheral satiety factor that inhibits feeding by regulating a variety of appetite-related hormones in the brain. However, most of the previous studies examining leptin in fish feeding were performed with mammalian leptins, which share very low sequence homologies with fish leptins. To elucidate the function and mechanism of endogenous fish leptins in feeding regulation, recombinant goldfish leptin-AI and leptin-AII were expressed in methylotrophic yeast and purified by immobilized metal ion affinity chromatography (IMAC). By intraperitoneal (IP) injection, both leptin-AI and leptin-AII were shown to inhibit the feeding behavior and to reduce the food consumption of goldfish in 2 h. In addition, co-treatment of leptin-AI or leptin-AII could block the feeding behavior and reduce the food consumption induced by neuropeptide Y (NPY) injection. High levels of *leptin receptor* (*lepR*) mRNA were detected in the hypothalamus, telencephalon, optic tectum and cerebellum of the goldfish brain. The appetite inhibitory effects of leptins were mediated by downregulating the mRNA levels of orexigenic *NPY*, *agouti-related peptide* (*AgRP*) and *orexin* and upregulating the mRNA levels of anorexigenic *cocaine-amphetamine-regulated transcript* (*CART*), *cholecystokinin* (*CCK*), *melanin-concentrating hormone* (*MCH*) and *proopiomelanocortin* (*POMC*) in different areas of the goldfish brain. Our study, as a whole, provides new insights into the functions and mechanisms of leptins in appetite control in a fish model.

## 1. Introduction

Leptin, the protein product of the *obese* gene, was first identified in mouse adipose tissue by positional cloning [1]. In mammals, leptin plays a major role as a satiety factor in feeding termination [2] by blocking the synthesis and secretion of orexigenic neuropeptide Y (NPY) [3] and agouti-related peptide (AgRP) [4] and promoting the expression and secretion of anorexigenic proopiomelanocortin (POMC) [5] and cocaine-amphetamine-regulated transcript (CART) [6] in the central nervous system. Leptin regulates appetite through a cell-surface leptin receptor (lepR) that is a member of the type I cytokine receptor family [7,8]. At least six transcripts from a single *lepR* gene have been reported to produce multiple lepR isoforms in mammals, but only the long form (lepRb) is responsible for the actions of leptin [9]. The mammalian *lepR* is expressed in a variety of brain regions, including the midbrain, hindbrain and hypothalamus [10]. In the hypothalamus in particular, lepR was found to be a crucial link between peripheral leptin and the central appetite regulators [11,12].

After cloning the *leptin* gene in mammals, it took more than a decade to identify the first non-mammalian *leptin* orthologues [13,14,15] due to the fact that there is very low sequence homology between the mammalian and non-mammalian leptins [16,17]. Because of the fish-specific genome duplication (FSGD or 3R) occurring in teleosts, duplicated *leptin* genes (*leptin*-A and *leptin*-B) have been reported in several fish species [18,19,20,21], whereas only a single *leptin* gene is found in mammals [16]. Based on both structural analysis [22] and post-receptor signaling assays [20], the receptor-binding affinity of fish leptin-A was found to be higher than that of leptin-B. Moreover, another genome duplication, called tetraploidization, in cyprinids [13] and salmonids [23] resulted in up to four leptin paralogues in these species [16]. In goldfish (*Carassius auratus*), two *leptin* genes (GenBank: FJ534535.1 and FJ854572.1) have been reported. They are phylogenetically clustered with other Cyprinidae *leptin*-A genes and were therefore named *leptin*-AI and *leptin*-AII, respectively [24].

As one of the most important peripheral appetite-inhibiting hormones described in mammals [25], leptin’s functions in feeding control have also been observed in fish models. Using both intraperitoneal (IP) and intracerebroventricular (ICV) injection, recombinant mouse leptin was shown to reduce food intake [26,27], body weight [27] and locomotor activity [28] in goldfish. The stimulatory effects of NPY and orexin on goldfish feeding may be blocked by co-injection with mouse leptin [26]. In addition, the inhibitory effects of CART and cholecystokinin (CCK) on goldfish feeding could be reinforced by co-injection with mouse leptin [26,29]. After the discovery of the teleostean *leptin* cDNAs, recombinant rainbow trout leptin-A protein was assessed, and its anorexigenic effects were confirmed [30]. In the hypothalamus of rainbow trout, mRNA expressions of *NPY* and *POMC* were decreased and increased, respectively, after injection of recombinant trout leptin-A [31]. In the *lepR*-deficient (knockout) medaka, NPY and AgRP mRNA levels were upregulated, while the POMC transcript level was downregulated [32].

**Figure 1:**
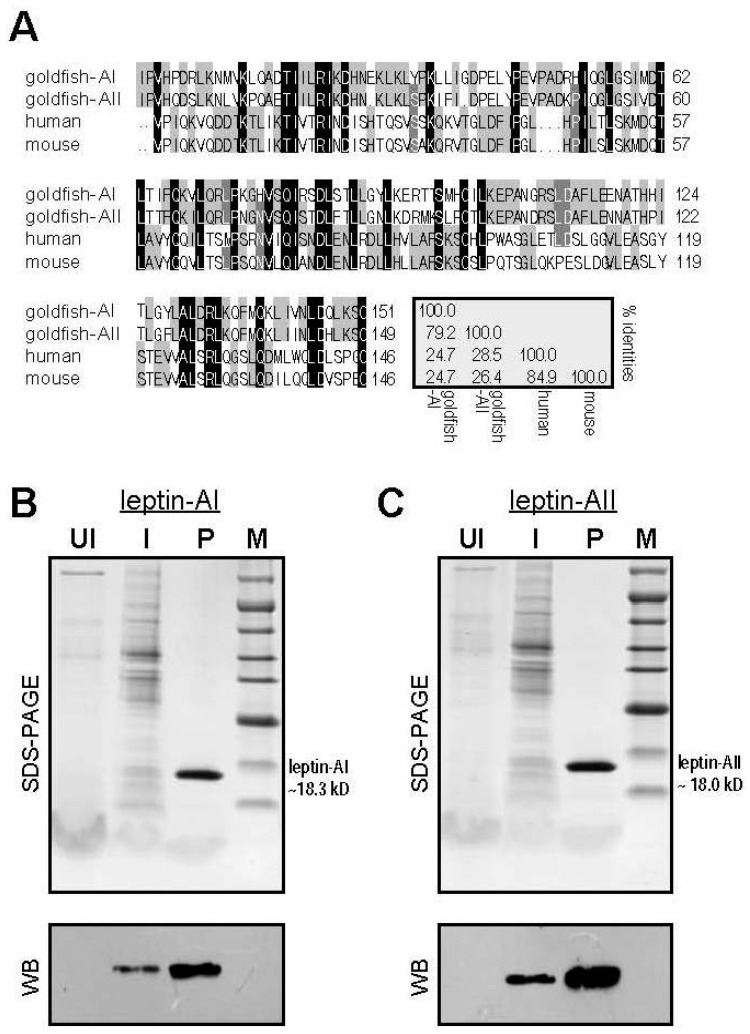
(**A**) Amino acid sequence alignment of goldfish leptin-AI, goldfish leptin-AII, human leptin and mouse leptin. The conserved amino acid residues are boxed in black, and similar amino acid residues are labeled in gray. The sequence identities among these four leptins are also listed; (**B**) Expression and purification of recombinant goldfish leptin-AI protein; (**C**) Expression and purification of recombinant goldfish leptin-AII protein. UI: the total supernatant of the culture without methanol induction; I: the total supernatant of the culture with methanol induction; P: isolated protein after Ni-column purification and PD-10 column desalting; M: protein marker. SDS-PAGE: sodium dodecyl sulfate polyacrylamide gel electrophoresis; WB: Western blot analysis.

Goldfish is a freshwater fish belonging to the family Cyprinidae under the order Cypriniformes. It has been well established as a model for feeding experiments [33]. Food intake of goldfish is controlled by a number of appetite regulators produced by the brain [34]. The central orexigenic factors, e.g., NPY [35], AgRP [36], orexin [37,38] and apelin [39], and the anorexigenic factors, e.g., CART [29,37], CCK [26], α-melanocyte-stimulating hormone (α-MSH, a protein product of *POMC*) [40] and melanin-concentrating hormone (MCH) [35], have been shown to regulate appetite in goldfish. The actions of leptin on appetite control were demonstrated independently [27,28] or cooperatively [26,29] in goldfish. However, previous studies examining the effects of leptin on goldfish feeding were all performed using mammalian leptins [26,27,28], which share only ~25% sequences identities with the endogenous goldfish leptin-AI or leptin-AII (Figure 1A). As reported in tilapia, mammalian leptin was unable to activate the fish lepR, in contrast to fish leptins [20], indicating that the previously reported mammalian leptin anorexigenic effects in fish are likely mediated via an unclear mechanism without activation of the central lepR. Thus, the actions and mechanisms of teleostean leptins on goldfish feeding still need to be evaluated. In this study, we first produced the goldfish leptin-AI and leptin-AII recombinant proteins in a eukaryotic methylotrophic yeast system. The actions of goldfish leptin-AI and leptin-AII on feeding behavior and food consumption were subsequently examined. The expression profile of *lepR* mRNA in different regions of the goldfish brain was determined. The mechanism for leptin-regulated feeding in goldfish was investigated by detecting the mRNA levels of various appetite-regulating neuropeptides in selected regions of the brain.

## 2. Results

### 2.1. Alignment of Goldfish and Mammalian Leptin Mature Peptide Sequences

An alignment was performed using the goldfish leptin-AI, leptin-AII, human and mouse leptin mature peptide a.a. sequences (Figure 1A). Conserved protein regions were found between goldfish leptin-AI and leptin-AII or between human leptin and mouse leptin. However, no conserved regions were detected between the goldfish leptins and the mammalian leptins. The sequence identity between goldfish leptin-AI and leptin-AII is high (79.2%). In contrast, the sequence identities between goldfish leptins and mammalian leptins are low (24.7%–28.5%).

### 2.2. Expression and Purification of Goldfish Leptin-AI and Leptin-AII Protein

Recombinant goldfish leptin-AI and leptin-AII were expressed as N-terminal His-tagged fusion proteins in *P. pastoris* X33 and purified by immobilized metal ion affinity chromatography (IMAC). SDS-PAGE followed by Western blotting with an anti-His-tag antibody showed that high-purity recombinant goldfish leptin-AI (~18.3 kD) and leptin-AII (~18.0 kD) proteins with immunoreactive His-tags were obtained (Figure 1B).

### 2.3. Effects of Leptin-AI and Leptin-AII on Goldfish Feeding Behavior and Food Consumption

**Figure 2:**
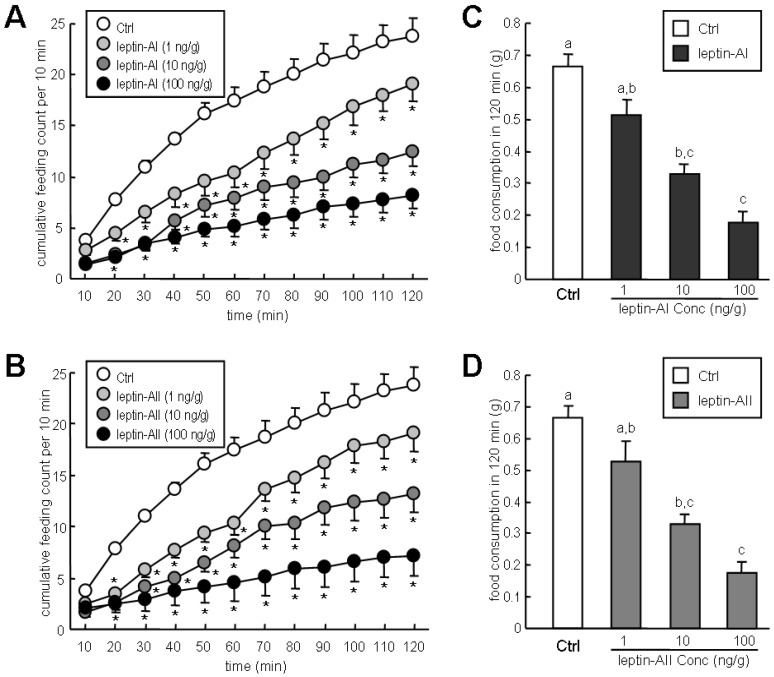

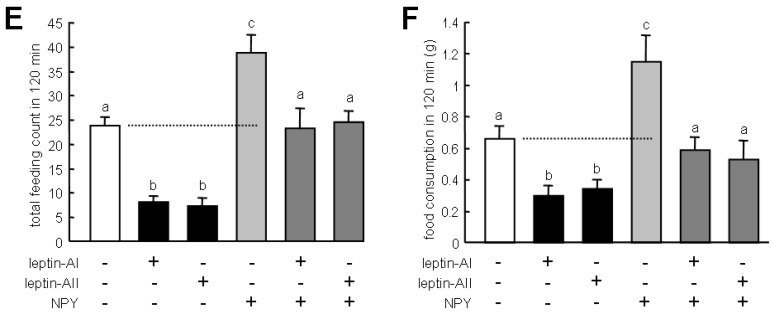
Time-dependent effects of leptin-AI (**A**) and leptin-AII (**B**) IP injection on feeding behavior in goldfish. Observational experiments for feeding were divided into 10-min periods and lasted for 2 h with fish injected with either physiological saline (*n* = 20), leptin-AI (1 ng/g bwt, *n* = 16; 10 ng/g bwt, *n* = 15; and 100 ng/g bwt, *n* = 16) or leptin-AII (1 ng/g bwt, *n* = 17; 10 ng/g bwt, *n* = 16; and 100 ng/g bwt, *n* = 15); Effects of leptin-AI (**C**) and leptin-AII (**D**) IP injection on food consumption in goldfish. Total amount of food consumed at 2 h was measured with fish injected with either physiological saline (*n* = 19), leptin-AI (1 ng/g bwt, *n* = 15; 10 ng/g bwt, *n* = 15; and 100 ng/g bwt, *n* = 16) or leptin-AII (1 ng/g bwt, *n* = 16; 10 ng/g bwt, *n* = 15; and 100 ng/g bwt, *n* = 15); Interaction of leptin-AI, leptin-AII and NPY IP injection on feeding behavior (**E**) and food consumption (**F**) in goldfish. For feeding behavior observation, the individual numbers in the control, leptin-AI only, leptin-AII only, NPY only, leptin-AI + NPY and leptin-AII + NPY groups were 20, 16, 15, 16, 15 and 15, respectively. For food consumption measurement, individual numbers of the control, leptin-AI only, leptin-AII only, NPY only, leptin-AI + NPY and leptin-AII + NPY groups were 19, 16, 15, 16, 15 and 15, respectively. In these studies, data are expressed as the mean ± SE. Significant differences between treated and untreated groups were examined by Student’s *t*-test (* *p* < 0.05) or one-way ANOVA (*p* < 0.05).

The effects of leptin-AI and leptin-AII on goldfish feeding behavior and food consumption were determined after IP injection. Injection of both recombinant leptin-AI (Figure 2A) and leptin-AII (Figure 2B) protein reduced feeding behavior of goldfish in dose-dependent manners (1–100 ng/g body weight (bwt)). The effects of leptin-AI and leptin-AII on goldfish feeding behavior were highly similar, and the maximum inhibitory effects of leptin-AI and leptin-AII were 65.9% (Figure 2A) and 70.0% (Figure 2B), respectively. Similarly, food consumption of goldfish in 2 h was reduced by IP injection of recombinant leptin-AI or leptin-AII in dose-dependent manners (1–100 ng/g bwt). The maximum reduction for leptin-AI (100 ng/g bwt) and leptin-AII (100 ng/g bwt) on food consumption were approximately to 28.1% (Figure 2B) and 26.4% (Figure 2D) of the basal level, respectively. In the same experiment, IP injection of NPY (50 ng/g bwt) increased the feeding behavior (74.0% of the basal level, Figure 2E) and food consumption (62.6% of the basal level, Figure 2F) in goldfish, but these stimulatory effects were abolished upon co-treatment with leptin-AI or leptin-AII (Figure 2E,F).

### 2.4. Expression Profile of Leptin Receptor in Different Brain Regions

**Figure 3:**
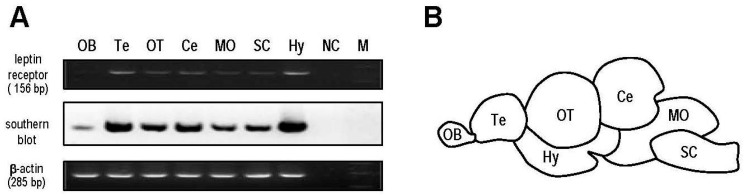
(**A**) Expression profile of goldfish *lepR* in different brain regions, including the olfactory bulb (OB), telencephalon (Te), optic tectum (OT), cerebellum (Ce), medulla oblongata (MO), spinal cord (SC) and hypothalamus (Hy), as assessed by semi-quantitative RT-PCR and subsequently confirmed by Southern blotting; (**B**) diagram showing the goldfish brain regions.

The mRNA distribution profile of *lepR* was examined semi-quantitatively in different areas of the goldfish brain (Figure 3A,B). Signals for *lepR* were observed in all brain regions. By a semi-quantitative approach, the highest transcript level of *lepR* was present in the hypothalamus, followed by the telencephalon, optic tectum and cerebellum, and low, but detectable levels of *lepR* were found in the medulla oblongata, spinal cord and olfactory bulb. The PCR results were further confirmed by Southern blotting.

### 2.5. Transcript Expression of Appetite-Related Hormones in Selected Brain Regions after Leptin-AI and Leptin-AII Injection

**Figure 4:**
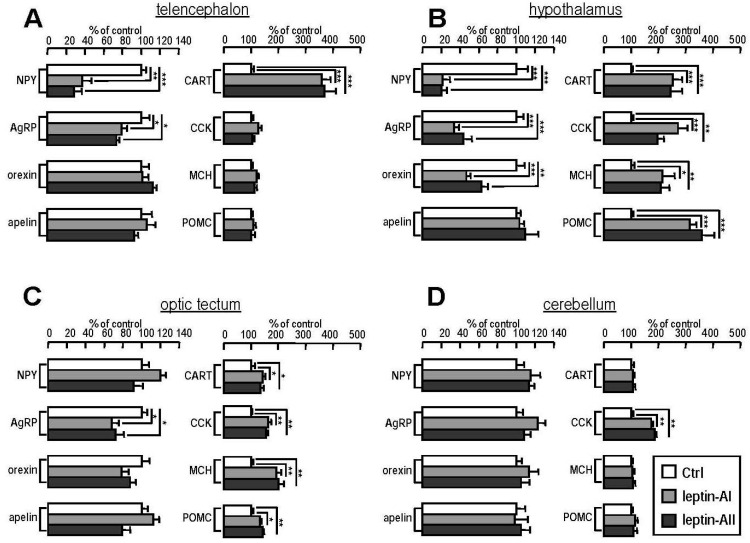
Regulation of leptin-AI and leptin-AII IP injection on orexigenic and anorexigenic factors expressed in selected brain regions in goldfish. The brain regions include the telencephalon (**A**), hypothalamus (**B**), optic tectum (**C**) and cerebellum (**D**). The detected orexigenic factors included *NPY*, *AgRP*, *orexin* and *apelin*, and the anorexigenic factors included *CART*, *CCK*, *MCH* and *POMC*. In this study, IP injection with FFPS was used as the control treatment, and real-time PCR of β*-actin* was used as the internal control. The data (*n* = 10) were expressed as a percentage of the control group. Data are expressed as the mean ± SE, and significant differences between treated and untreated groups were assessed by Student’s *t*-test (* *p* < 0.05, ** *p* < 0.01 and *** *p* < 0.001).

The effects of leptin-AI and leptin-AII on the expression of appetite-related hormones, such as *NPY*, *AgRP*, *orexin*, *apelin*, *CART*, *CCK*, *MCH* and *POMC*, were examined in different brain regions, including the telencephalon (Figure 4A), hypothalamus (Figure 4B), optic tectum (Figure 4C) and cerebellum (Figure 4D). For orexigenic factors, at 2 h after IP injection of leptin-AI or leptin-AII, *NPY* levels were downregulated in the telencephalon and hypothalamus; *AgRP* levels were downregulated in the telencephalon, hypothalamus and optic tectum; *orexin* levels were downregulated only in the hypothalamus; and apelin levels did not change in any brain regions. For anorexigenic factors, at 2 h after IP injection of leptin-AI or leptin-AII, *CART* levels were upregulated in the telencephalon, hypothalamus and optic tectum; *CCK* levels were upregulated in the hypothalamus, optic tectum and cerebellum; *MCH* levels were upregulated in the hypothalamus and optic tectum; and *POMC* levels were upregulated in the hypothalamus and optic tectum. These results indicate that the actions of leptins on feeding control in goldfish (demonstrated by the feeding behavior observations and food consumption measurements) were likely mediated by upregulation of several orexigenic factors (e.g., NPY, AgRP and orexin) and the downregulation of several anorexigenic factors (e.g., CART, CCK, MCH and POMC).

## 3. Discussion

Previous studies evaluating leptin regulation of feeding in fish were performed with mammalian leptins [26,27,28], except for one report that demonstrated that IP injection of recombinant teleostean leptin suppressed food intake in rainbow trout [30]. The fish leptins are similar to the mammalian leptins in three-dimensional (3-D) structure, but distant in the primary amino acid sequences [14,16]. A study in tilapia showed that mouse leptin cannot promote STAT-3 signaling in the tilapia lepR-transfected COS7 cells, and STAT-3 signaling is necessary for leptin regulation of appetite [41]. However, another study in tilapia demonstrated that the effects of human and tilapia leptin on *prolactin* gene expression were similar [42]. Therefore, the effects of mammalian leptins on feeding in fish, including those in goldfish [26,27,28], may be mediated via an unknown mechanism instead of the classical lepR/STAT-3 pathway in mammals. In this study, the goldfish leptin-AI and leptin-AII proteins were expressed in a eukaryotic methylotrophic yeast system for functional studies to determine whether goldfish leptins have anorexigenic effects similar to their mammalian counterparts [26,28]. Functional recombinant proteins of non-mammalian leptins from clawed frog [15], rainbow trout [30] and tilapia [20,42] have been produced in prokaryotic *Escherichia coli* systems. In contrast, we failed to express goldfish leptin-AI and leptin-AII in an *E. coli* system with any of the expression vectors and bacterial strains that were used. Nevertheless, our current study is the first report for the generation of a non-mammalian leptin protein using a eukaryotic methylotrophic yeast system. The first mammalian leptin protein produced in methylotrophic yeast was ovine leptin [43].

Using an IP injection approach, we found that both recombinant leptin-AI and leptin-AII could reduce the frequencies of feeding behavior (Figure 2A,B) and the food consumption (Figure 2C,D) of goldfish in time- and dose-dependent manners. In contrast to previous studies using mammalian leptins [26,27,28], our current study is the first to demonstrate the anorexigenic effects of leptin in goldfish using goldfish leptins. Similar inhibitory effects of non-mammalian leptins on food consumption have also been reported in frog [15] and rainbow trout [30]. In addition, IP injection of NPY, the most potent orexigenic factor in fish [26,44], increased feeding behavior and food consumption in goldfish, but its orexigenic effects were blocked by co-injection of goldfish leptin-AI or leptin-AII (Figure 2E,F). These results are similar to a previous study that ICV injection of mouse leptin could inhibit NPY-induced feeding in goldfish [26]. Given that IP injection of leptins could block NPY induction of feeding, it was logical to conclude that the goldfish leptins act on the central nervous system (CNS) and block the stimulatory effects induced by orexigenic factors in feeding (e.g., NPY). Furthermore, the anorexigenic effects of leptin-AI and leptin-AII were highly comparable (Figure 2). The differentiation of leptin-AI and leptin-AII during Cypriniformes genome tetraploidization is estimated to have occurred only ~16 million years ago (Mya) [45], which is much more recent than the divergence of mammalian and fish leptins (~450 Mya) [46] and of fish leptin-A and leptin-B (~296 Mya) [47]. Therefore, the a.a. sequence homology shared between leptin-AI and leptin-AII is relatively high (e.g., 79.2% in goldfish, as shown in Figure 1A), and the feeding inhibition effects of leptin-AI and leptin-AII were also similar.

High expression levels of *lepR* mRNA have been reported in the CNS of goldfish [24]. Using semi-quantitative RT-PCR coupled with Southern blotting, it was shown that the telencephalon, hypothalamus, optic tectum and cerebellum were the major sites for *lepR* mRNA expression in the goldfish brain (Figure 3). The mechanism for leptin regulation of appetite in goldfish was determined by detecting the changes in several appetite regulator transcripts in different brain regions. Given that most of the feeding behaviors and food consumption of goldfish occurred in the first 2 h after food administrated (Figure 2A–D), we selected 2 h after leptin injection as the time point to measure the expression of brain appetite regulators. In this case, leptin-AI and leptin-AII were found to downregulate the expression of the orexigenic hormones *NPY*, *AgRP* and *orexin* and upregulate the expression of the anorexigenic hormones *CART*, *CCK*, *MCH* and *POMC* (Figure 4). The hypothalamus may be the most important brain region for the leptin action on appetite in goldfish, based on the fact that leptin administration could alter the expression levels of seven appetite regulators in this area (Figure 4B). In the forebrain (telencephalon), leptin was more effective at inhibiting appetite stimulator transcripts (Figure 4A), while in the hindbrain (optic tectum and cerebellum), leptin was more effective at stimulating appetite inhibitor transcripts (Figure 4C,D). However, different central appetite regulators (e.g., NPY, orexin, apelin, CART and MCH) may influence each other within the goldfish brain [34]. Therefore, the effects of leptin on central appetite regulators in the goldfish brain may be either via a direct or an indirect mechanism. In mammals, leptin suppressed food intake mediated by NPY/AgRP and POMC/CART neurons in arcuate nucleus, the hypothalamic feeding center of mammals [48]. Mediation of the anorexigenic activity of leptin by central *NPY* and *POMC* has also been reported in rainbow trout [30]. Additionally, ICV injection of mouse leptin increased the hypothalamic *CCK* mRNA expression in goldfish [26]. These results, along with our study, indicate that the neuroendocrine pathways of leptin in feeding control are conserved from teleosts to mammals.

## 4. Materials and Methods

### 4.1. Animals

Sexually immature goldfish (*Carassius auratus*) from the same family with body weights ranging from 25–30 g and body lengths ranging from 12 to 15 cm were acquired from local suppliers and maintained individually in 9-L tanks at 20 to 25 °C under a 12:12 h dark-light photoperiod. Prior to the feeding experiments, goldfish were acclimated to the feeding schedule (1 g per fish, once every day at 10:00 A.M.) for 14 days with fish food in the form of pre-dried floating pellets. For gene expression studies, goldfish were sacrificed by spinosectomy after anesthesia with 0.05% tricaine methanesulfonate (MS222, Sigma, St. Louis, MO, USA) according to the guidelines and approval of the Ethics Committees of the South China Sea Institute of Oceanology, Chinese Academy of Sciences.

### 4.2. Sequence Alignment and Identities Analysis

The mature peptide amino acid (a.a.) sequences of goldfish leptin-AI (GenBank: ACL68083.1), leptin-AII (GenBank: ACO82076.1), human leptin (GenBank: NP_000221.1) and mouse leptin (GenBank: NP_032519.1) were compared. The sequence alignment was performed using Clustalx1.8 and presented with GeneDoc. The sequence identities were calculated using MegAlign.

### 4.3. Production of Goldfish Leptin-AI and Leptin-AII Recombinant Protein in P. pastoris

Recombinant goldfish leptin-AI and leptin-AII proteins were generated in the methylotrophic yeast (*Pichia pastoris*) system following a previously described protocol with modifications [49]. Briefly, the sequences encoding the mature proteins of goldfish *leptin*-AI (GenBank: FJ534535.1) and *leptin*-AII (GenBank: FJ854572.1) were amplified by PCR, and eight histidines (8× His-tag) were added at the N-terminus of the cDNA sequences. The purified PCR products were subsequently sub-cloned into the pPICZ*α*A vector (Invitrogen, Grand Island, NY, USA). The plasmids were linearized with *Sac*I (TaKaRa, Kusatsu, Japan) and transformed into *P. pastoris* X33 cells (Invitrogen) by electroporation. Positive transformants were selected on yeast extract peptone dextrose (YPD) plates with 500 μg/mL zeocin (Invitrogen). Then, positive clones were cultured in 10 mL buffered glycerol complex medium (BMGY) at 28 °C for 24 h for the growth phase and were then grown in buffered methanol complex medium (BMMY) at 28 °C for another 3 days with the addition of 1% methanol each 24 h for induction. The best clones secreting recombinant protein were used for large-scale production by flask culture. Both goldfish leptin-AI and leptin-AII proteins were purified by His-Bind Kits (Novagen, Darmstadt, Germany) and desalted with PD-10 desalting columns (GE Healthcare, Chicago, IL, USA). The purity and identity of the recombinant proteins were determined by SDS-PAGE and Western blot analysis as described previously [50]. The concentration of goldfish leptin-AI and leptin-AII recombinant protein was determined by the bicinchoninic acid (BCA) method (Sangon, Shanghai, China).

### 4.4. Effect of Leptin-AI and Leptin-AII on Goldfish Feeding Behavior and Food Consumption

The effects of leptin-AI and leptin-AII on the appetite of goldfish were analyzed following IP injection. After deep anesthesia with 0.05% MS222, 100 μL of protein solution (leptin-AI or leptin-AII, dissolved in freshwater fish physiological saline (FFPS) [51], at final concentrations of 1, 10 and 100 ng/g body weight (bwt)) was injected into the peritoneal cavity by a 23-gauge needle attached to a 1-mL syringe, and injection of FFPS only was used as a control. The interaction of leptins and orexigenic factors was demonstrated by co-injection of NPY (50 ng/g bwt, Tocris Bioscience, Bristol, UK) with leptin-AI (100 ng/g) or leptin-AII (100 ng/g), and injection of NPY (50 ng/g bwt) only was used as a positive control. After injection, the fish was allowed to recover from anesthesia by flushing fresh water over the gills. A single fish was placed in an individual 9-L tank for observation of feeding behavior, and 1.5 g of dry floating food pellets were administered per observation session for a total of 2 h. Feeding behavior was assessed by counting the number of feeding acts as described by Volkoff and Peter [52]. Then, the uneaten food pellets in each tank were recovered. After drying completely, the dry mass of the uneaten food pellets was weighed and recorded. The amount of food consumed was estimated by subtracting the amount of food remaining (uneaten) at the end of the experiment from the total amount of food administered.

### 4.5. Semi-Quantitative Analysis of Leptin Receptor Expression in Different Brain Regions

The expression profiles of *lepR* in various brain regions of the goldfish were examined using semi-quantitative RT-PCR. Briefly, total RNA was isolated using TRIzol (Invitrogen) from selected brain areas, including the olfactory bulb, telencephalon, optic tectum, cerebellum, medulla oblongata, spinal cord and hypothalamus. The RNA samples were digested with DNase I (Invitrogen) and reverse transcribed using a PrimeScript™ RT kit (TaKaRa). The RT samples were then used as the templates for PCR using primers specific for goldfish *lepR* with amplified cycle numbers of 35 based on semi-quantitative validation. Then, the PCR products were resolved in a 2% gel, visualized by staining with ethidium bromide, and transblotted onto a positively-charged nylon membrane. To assess the identity of PCR products, a Southern blot was conducted using a DIG-labeled cDNA probe for goldfish *lepR* as described previously [53]. In this case, RT-PCR for β*-actin* was also performed to serve as an internal control.

### 4.6. Effects of Leptin-AI and Leptin-AII on Gene Expression of Appetite Regulators in Different Brain Regions

Goldfish leptin-AI (100 ng/g bwt) or leptin-AII (100 ng/g bwt) recombinant proteins were administered by IP injection, with parallel treatment of FFPS as a control. After injection, goldfish were allowed to recover from MS222 anesthesia for 5 min. Fish were sacrificed, and the telencephalon, optic tectum, cerebellum and hypothalamus were harvested at 2 h following the 5-min recovery period. The samples were frozen in liquid nitrogen and stored at −80 °C for RNA extraction and reverse transcription. The mRNA expression of orexigenic factors (*NPY*, *AgRP*, *orexin* and *apelin*) and anorexigenic factors (*CART*, *CCK*, *MCH* and *POMC*) in the forebrain were detected by real-time PCR.

### 4.7. Measurement of Transcriptional Expression of Target Genes by Real-Time PCR

Total RNA from tissue samples was isolated using TRIzol, digested with DNase I and reverse transcribed with a PrimeScript™ RT kit. Transcriptional expression of target genes (*NPY*, *AgRP*, *orexin*, *apelin*, *CART*, *CCK*, *CART*, *MCH*, *POMC* and β*-actin*) was detected using SYBR Premix Ex Taq™ II (TaKaRa) in a Rotor-Gene RG-3000 real-time PCR system (Qiagen, Duesseldorf, Germany) with primers and PCR conditions as reported previously (Table 1) [54]. Serially-diluted plasmid DNAs containing ORF sequences for the target genes were used as the standards for real-time PCR. After PCR reactions, the identities of the PCR products were routinely confirmed by melting curve analysis.

### 4.8. Data Transformation and Statistical Analysis

For real-time PCR, the raw data of target gene expression were expressed as fmol per tube and routinely normalized as a ratio of β*-actin* mRNA detected in the same sample. Given that no significant differences were detected for β*-actin* expression in our experiments, the raw data of target gene expression were simply transformed as a percentage of the mean values of the control group for statistical analysis. Data were expressed as the mean ± SE and analyzed by Student’s *t*-test or one-way ANOVA followed by Fisher’s least significant difference (LSD) test with SPSS (IBM Software, Armonk, NY, USA). The significant differences were considered at *p* < 0.05.

## 5. Conclusions

In conclusion, we report the first functional recombinant production of goldfish leptin-AI and leptin-AII from a eukaryotic methylotrophic yeast system. The anorexigenic effects of leptin-AI and leptin-AII in goldfish were confirmed by the inhibition of feeding behavior and reduced food consumption. The mechanism of leptin-regulated appetite was determined by assessing a group of central orexigenic and anorexigenic regulators in different brain regions. Our study, as a whole, provides new insight into the function and mechanism of leptins in appetite control in a fish model.

## Figures and Tables

**Table 1:** Primers and amplification conditions for PCR analysis in this study.

Gene Target/Accession No. (Primer Sequences, 5′-3′)	PCR Condition				Cycle	*T*_m_	Product Size
	Denaturing	Annealing	Extension	Detection			
***NPY*/M87297**							
GTAGTGTTGCGGGTAGCGA	94 °C	64 °C	72 °C	88 °C	×35	92 °C	234 bp
CAGACACCCCGACCCAAG	30 s	30 s	30 s	20 s			
***AgRP*/AJ555492**							
TGGCATCACATCCAAACCT	94 °C	64 °C	72 °C	82 °C	×35	88 °C	230 bp
CAGGTGATGACCCAAGCAG	30 s	30 s	30 s	20 s			
***Orexin*/DQ923590**							
GCAGAGCTGCTCATTGTTGACGTT	94 °C	64 °C	72 °C	84 °C	×35	82 °C	286 bp
AACCTTGTGATTACCTCAGGAGT	30 s	30 s	30 s	20 s			
***Apelin*/FJ755698**							
GAGCATAGCAAAGAGCTGGA	94 °C	64 °C	72 °C	89 °C	×35	94 °C	340 bp
GCTGAGGATGAGTGGCTTGT	30 s	30 s	30 s	20 s			
***CART*/AF288810**							
CCAAAGGACCCGAATCTGA	94 °C	64 °C	72 °C	82 °C	×35	90 °C	171 bp
TTTGCCGATTCTTGACCCT	30 s	30 s	30 s	20 s			
***CCK*/CAU70865**							
CCGCAGTCTCAGAAGATGGG	94 °C	64 °C	72 °C	87 °C	×35	91 °C	197 bp
GGAGGGGCTTCTGCGATA	30 s	30 s	30 s	20 s			
***MCH*/AM403730**							
AGGCTTGAGCGAGAACTTGG	94 °C	64 °C	72 °C	86 °C	×35	91 °C	272 bp
CCCAGAAGACCTACACCTCCC	30 s	30 s	30 s	20 s			
***POMC*/AJ431209**							
AAGCGCTCCTACTCCATGGA	94 °C	60 °C	72 °C	83 °C	×35	85 °C	282 bp
CTCGTCCCAGGACTTCATGAA	30 s	30 s	30 s	20 s			
**β*-actin*/AB039726**							
CTGGTATCGTGATGGACTCT	94 °C	56 °C	72 °C	87 °C	×35	91 °C	285 bp
AGCTCATAGCTCTTCTCCAG	30 s	30 s	30 s	20 s			
